# Particle Filtering for Three-Dimensional TDoA-Based Positioning Using Four Anchor Nodes

**DOI:** 10.3390/s20164516

**Published:** 2020-08-12

**Authors:** Mohamed Khalaf-Allah

**Affiliations:** Institute of Traffic Telematics, Technische Universität Dresden, 01062 Dresden, Germany; mohamed.khalaf_allah@tu-dresden.de; Tel.: +49-351-463-37556

**Keywords:** time difference of arrival (TDoA), hyperbolic positioning, particle filtering, unmanned air vehicle (UAV), drone, indoor positioning

## Abstract

In this article, the four-anchor time difference of arrival (TDoA)-based three-dimensional (3D) positioning by particle filtering is addressed. The implemented particle filter uses 1000 particles to represent the probability density function (pdf) of interest, i.e., the posterior pdf of the target node’s state (position). A resampling procedure is used to generate particles in the prediction step, and TDoA measurements are used to determine the importance, i.e., weight, of each particle to enable updating the posterior pdf and estimating the position of the target node. The simulation results show the feasibility of this approach and the possibility to employ it in indoor positioning applications under the assumed working conditions using, e.g., the ultra-wideband (UWB) wireless technology. Therefore, it is possible to enable unmanned air vehicle (UAV) positioning applications, e.g., inventory management in large warehouses, without the need for an excessive number of anchor nodes.

## 1. Introduction

The localization of a target node using time difference of arrival (TDoA) measurements received and is still receiving considerable attention. TDoA-based positioning algorithms [1,2,3,4,5,6,7] are widely applied in sensor networks [1,8], wireless communication [2,3], target tracking [9,10], navigation [11], underwater [12], tactile interfacing for human–computer interaction [13], and seismic exploration [14]. The determination of a unique target node’s location using TDoA measurements in closed form requires at least four anchor nodes, i.e., three TDoA measurements, in the two-dimensional (2D) space and five anchor nodes, i.e., four TDoA measurements, in the three-dimensional (3D) space [15]. However, an object’s location can also be determined with one fewer anchor node, i.e., three and four anchor nodes for the 2D and 3D spaces, respectively, if further information is available to resolve any location ambiguities [16]. Solving the TDoA-based 2D/3D positioning problem with an iterative algorithm using three/four anchor nodes requires a good initial position estimate to guarantee convergence to the true position.

Unmanned air vehicles (UAVs), also known as drones, are an emerging technology with a surge in many different fields of application [17]. Both expressions (UAV and drone) are used interchangeably throughout the article. Recent technological advancements increased the functionalities and features of drones, while unit prices are constantly decreasing. Drones can be equipped with various sensors to achieve certain functions. Sensors for guidance and control functions include positioning sensors, e.g., global navigation satellite system (GNSS) receivers, inertial sensors, e.g., accelerometers and gyroscopes, barometers, digital compasses, and ultrasound sensors. Communication functions are accomplished using radio wave antennas of different frequency, such as WLAN, Wi-Fi, RFID, ultra-wideband (UWB), and 5G. Environmental monitoring tasks are performed using video cameras, LiDAR sensors, microphones, speakers, IR and light sensors, proximity sensors, magnetic sensors, temperature sensors, chemical sensors, gas sensors, etc.

The employment of drones for emergency response is an important field of application [18,19]. Environmental mapping is another field of application [19], in which drones equipped with LiDAR sensors perform 3D spatial models of geographic areas, certain structures, and buildings. Drones equipped with video cameras are used to perform surveillance tasks during public events, as well as police, security, and military operations, in addition to recreational and leisure activities [19,20]. Logistics and transportation are two potential fields of application. Drones can be employed in delivery tasks such as package delivery and delivery of medicines and other materials during humanitarian and crisis management operations. There is a high interest in deploying robotic/mobile sensor networks for environmental monitoring [20]. In Reference [21], an aquatic microbial system was formed using a robotic boat and stationary buoys. An inexpensive, low-power, robotic fish was used in Reference [22] to reconstruct a spatiotemporal aquatic field. A mobile platform for aquatic environmental monitoring was developed in Reference [23]. A robotic boat with a depth sensor [24] sampled the depth of a lake. Autonomous underwater vehicles (AUVs) are important tools in oceanography, marine biology, and other maritime applications [25,26,27].

High-mobility and low-cost drones achieve larger coverage of ground objects [28,29] and, therefore, can be used for communication relaying, traffic control, and search and rescue applications [30]. Drones can also support the internet of vehicles (IoV) and other autonomous vehicle applications such as intelligent traffic management systems, automatic pilot, and formation control [28], where low-latency and high-accuracy positioning is highly demanded [31]. In Reference [28], UAVs were used to aid ground vehicle localization by broadcasting ranging measurements made with ground base stations and other UAVs. Each vehicle then estimates its position based on the broadcasted data and TDoA measurements from the UAVs, i.e., UAVs serve as anchor nodes for the vehicles. Drones are closer than satellites to objects on the ground and provide line-of-sight (LoS) links in most scenarios and, thus, deliver measurements with a higher signal-to-noise ratio (SNR), which is attractive for, e.g., autonomous vehicle high-accuracy localization [28].

The focus in this study is on 3D positioning of UAVs for indoor autonomous flight, where TDoA measurements from only four anchor nodes are used. The TDoA-based position solution provides long-term correction for UAV attitude estimation biases, which is essential for UAV active attitude control. A particle filtering approach is proposed to solve this hyperbolic, i.e., TDoA-based, positioning problem using four anchor nodes in the 3D space. An optimization algorithm to solve this problem under certain conditions was presented in Reference [16]. The proposed positioning method is a good option to be used with other sensors and technologies in an integrated framework. Flight control, mission, and path planning are beyond the scope of this study.

The main contributions of this article are (1) proposal of a particle filtering approach for the 3D positioning problem based on TDoA measurements from only four anchor nodes, and (2) performance evaluation of the proposed approach using simulation studies for UAV indoor positioning applications.

The rest of the article is organized as follows: Section 2 reviews recent works conducted on UAV indoor positioning. The particle filtering approach and the implemented algorithm are described in Section 3. Simulation results are presented in Section 4, and conclusions are given in Section 5.

## 2. Indoor Positioning of Unmanned Air Vehicles (UAVs)

The availability of long-life batteries, miniaturization, integration of various sensors, and increasing cost-effectiveness are making UAVs more attractive and affordable for civilian applications [32]. Therefore, many research efforts are devoted to exploring positioning solutions for indoor UAVs due to large market opportunities. Indoor UAV applications include tasks such as monitoring, inspection, surveillance, logistics, transportation, exploration, and mapping, in addition to location-based services (LBS) such as ambient assisted living, emergency response, gaming, social networking, advertisement, and entertainment. The indoor use of drones allows access to difficult-to-reach spaces and acceleration of data collection.

Indoor environments are much better protected from wind, wind gusts, fog, and rain than outdoor environments, thus making flying much easier [33]. Therefore, UAV indoor applications are becoming more attractive. To fly indoors, UAVs have to be small in size and able to fly slowly. A small-size rotorcraft UAV suits indoor applications very well because it can take off and land without user assistance and it requires only a limited area for takeoff and landing. Moreover, a rotorcraft UAV can maintain a constant 3D position and can approach the desired location very slowly, which are important features for performing desired tasks accurately.

Rotorcrafts require active attitude control because they are inherently unstable [33]. An inertial measurement unit (IMU) and autopilot can provide active attitude control only in the short term. In the long term, velocity or position measurements can correct the attitude estimation biases. Velocity information can be provided by a combination of visual measurements and additional sensors [34,35,36].

Surveys of UAV positioning and navigation in indoor and GNSS-denied environments were considered in References [32,37]. The ultrasonic technology is low-cost and can achieve centimeter-level accuracy if impairment factors are well compensated. In Reference [32], the performance of an ultrasonic local positioning system based on distance differences, i.e., TDoA measurements, was evaluated for UAV 3D indoor positioning. The obtained accuracies along the *x*-, *y*-, and *z*-coordinates were respectively 9, 5, and 28 cm using five ultrasonic anchors installed on the ceiling. A 3D wall following system based on five ultrasonic anchors and IMU measurements were implemented in Reference [38]. In Reference [39], ultrasonic-based 2D multilateration was used to determine the horizontal position of a vehicle, where the initial vertical position of the vehicle was obtained using a time of flight (ToF) camera. Other technologies for indoor positioning of drones include visible light communications (VLC) [40] and LiDAR [41,42].

Radiofrequency (RF) measurements are widely employed for indoor positioning since many RF systems are already deployed and are part of the communication infrastructure. RF wireless technologies used for positioning include WLAN or Wi-Fi, RFID, UWB, Bluetooth, ZigBee, and LTE. UWB is a high-bandwidth communication technology with multipath robustness and good material penetrability that can achieve centimeter-level accuracy for 3D indoor positioning. However, performance can be degraded under strong scattering conditions. UWB systems usually utilize time-based measurements such as time of arrival (ToA) or TDoA for position estimation. The work in Reference [43] utilized UWB TDoA measurements from eight anchor nodes for UAV indoor localization, in addition to optical flow and IMU sensors for velocity and yaw angle measurements, and less than 10 cm third quartile alignment errors were achieved. Monte Carlo localization (MCL) was used in Reference [44] to integrate visual odometry from RGB-D sensors with distance estimates to UWB sensors, considering a previously built multi-modal 3D map that contains the estimated UWB sensor locations, for UAV localization. The visual odometry provided the short-term attitude estimations, and the UWB measurements were used to correct the drift in the attitude estimates. Experiments in an indoor scenario of 15 × 15 × 5 m achieved positioning accuracies below 22 cm, where the millimeter-accurate ground truth was provided by a motion tracking system.

An extended Kalman filter (EKF) was utilized in Reference [45] to integrate IMU measurements and UWB range measurements from six anchors for 3D localization at a rate of 80 Hz. The proposed system was applied to a swarm of six micro aerial vehicles (MAVs) performing a light show in an indoor exhibition hall. In Reference [46], an inertial navigation system (INS) combined with UWB was used as an integrated positioning system for multi-quadrotor positioning and obstacle avoidance. Position estimates were obtained via an unscented Kalman filter (UKF). INS acceleration measurements were used for state propagation in the prediction step, and UWB range measurements from three anchors were utilized in the update step. Simulation results of two quadrotors were presented.

The need for automated inventory management solutions was recently increased to replace current manual methods [47]. UAVs were used in Reference [48] for inventory inspection at indoor warehouses to increase the efficiency of cost and time, as well as the safety of human workers. A low-cost sensing system was developed with an EKF-based multi-sensor fusion framework for autonomous navigation of UAVs in warehouse indoor environments. The used low-cost sensors included three cameras (forward, upward, and downward), a 2D laser scanner, a 1D range sensor, and an IMU, in addition to a map containing pose information of attached tags. The use of UAVs in warehouse inventory and similar applications was also reported in References [47,49,50,51,52,53].

## 3. Particle Filtering for TDoA-Based Positioning

Bayesian inference approaches provide well-studied tools for calculating the distribution of a target node’s location given TDoA measurements. Thus, the required probability density function (pdf) is the probability of the target node’s location given the available TDoA measurements. Bayes’ rule defines a method to calculate this pdf using prior information, a system or dynamic model, i.e., knowledge of target node’s motion, to describe the evolution of the target node’s location with time, and a measurement model relating noisy TDoA measurements to the location of the target node. Bayes’ rule is mathematically expressed as

(1) $$p\left(x|z\right)=\frac{p\left(z|x\right)p\left(x\right)}{\int p\left(z|x^{\prime }\right)p\left(x^{\prime }\right)dx^{\prime }},$$

where **x** is the state vector, i.e., the 3D location of the target node, which is a random variable; **z** is the noisy TDoA measurement vector; *p*(**x**) is the prior pdf of the state; *p*(**z**|**x**) is the measurement model, i.e., the pdf of the TDoA measurements conditioned on the state; *p*(**x**|**z**) is the posterior pdf of the state (pdf of interest), which describes the distribution of the target node’s location taking into account the TDoA measurements.

If the system and measurement models are both linear and Gaussian, the state can be optimally estimated in closed form using the Kalman filter (KF). If either the system or measurement model is nonlinear or non-Gaussian, the KF can only provide a suboptimal estimate to the non-Gaussian posterior pdf. Therefore, approximate computational methods are needed, which are based on the state space approach to time series modeling [54]. The approach adopted in this study proceeds in two steps (or stages): prediction and update. The prediction step propagates the state pdf at one time to the next time by using the dynamic model. Since the state is subject to unknown disturbances, modeled as random noise, the propagated state pdf is broadened and deformed. The last available measurements are used in the update step to modify, i.e., to tighten, the predicted state pdf. This is accomplished by using Equation (1), which mathematically describes the mechanism for updating knowledge about the state pdf in the light of new measurements.

### 3.1. Problem Statement and Concept of Solution

In nonlinear filtering problems, the state vector is denoted $x\left(t\right)\in {\mathbb{R}}^{3}$, where t is continuous-valued time. The state evolution is described using a continuous-time stochastic differential equation, also referred to as an Itô differential equation [55]. However, the state is usually sampled at discrete time instants and, thus, the state at the *k-*th discrete sample time is denoted $x_{k}\equiv x\left(t_{k}\right)$. The state evolves according to a continuous-time stochastic model, i.e., system model.

(2) dx(t)=f(x(t),dv(t),t,dt),

where f(·) is a known (possibly nonlinear) function of the state, and v(t) is a process noise sequence, which accounts for random disturbances in the target node’s motion.

TDoA measurements are a nonlinear function of the state, i.e., the target node’s location. They occur at times $t_{k}$, where k=1,2,…K. The *k-*th measurement is denoted $z\left(t\right)\in {\mathbb{R}}^{m}$, where *m* is the dimension of the measurement vector, i.e., the number of TDoA measurements available at any time instant $t_{k}$. If four anchor nodes are used, *m* = 3. The measurements are related to the state via the equation, i.e., measurement model.

(3) $$z_{k}=h_{k}\left(x_{k},w_{k}\right),$$

where $h_{k}\left(\cdot \right)$ is a known (possibly nonlinear) function, and $w_{k}$ is a measurement noise sequence.

Defining the measurement history $Z_{k}≜\left\{z_{1},\ldots z_{k}\right\}$, we seek estimates of $x_{k}$ based on the sequence of all available measurements up to the time $t_{k}$, i.e., the problem, from a Bayesian perspective, is to recursively calculate the posterior pdf $p(x_{k}|Z_{k})$ in two stages: prediction and update. The prediction step moves the pdf $p(x_{k-1}|Z_{k-1})$ forward in time to the pdf $p(x_{k}|Z_{k-1})$ using the system model and without incorporating the new TDoA measurements $z_{k}$. The update step incorporates the new TDoA measurements $z_{k}$, using the measurement model, into the predicted pdf $p(x_{k}|Z_{k-1})$ to obtain the updated, i.e., posterior, pdf $p(x_{k}|Z_{k})$.

The prediction step uses the system model in Equation (2) to obtain the predicted pdf $p(x_{k}|Z_{k-1})$ via the Chapman–Kolmogorov equation.

(4) $$p\left(x_{k}|Z_{k-1}\right)=\int p(x_{k}|x_{k-1},Z_{k-1})p(x_{k-1}|Z_{k-1})dx_{k-1}.$$

Equation (4) is a statement of the law of total probability. Since Equation (2) describes a first-order Markov process, the first term in the integral of Equation (4) simplifies to $p(x_{k}|x_{k-1})$, which is the probabilistic model of the state evolution. Thus, Equation (4) is rewritten as

(5) $$p\left(x_{k}|Z_{k-1}\right)=\int p(x_{k}|x_{k-1})p(x_{k-1}|Z_{k-1})dx_{k-1}.$$

The prediction, i.e., prior, pdf is updated by incorporating the available TDoA measurements $z_{k}$ to obtain the posterior pdf of the state via Bayes’ rule.

(6) $$p\left(x_{k}|Z_{k}\right)=p\left(x_{k}|z_{k},Z_{k-1}\right)=\frac{p\left(z_{k}|x_{k},Z_{k-1}\right)p\left(x_{k}|Z_{k-1}\right)}{p\left(z_{k}|Z_{k-1}\right)}.$$

The pdf on the denominator is the normalizing constant. The first term on the nominator, i.e., the (measurement) likelihood function, which is defined by the measurement model in Equation (3), simplifies to $p\left(z_{k}|x_{k}\right)$ due to conditional independence. Thus, Equation (6) is rewritten as

(7) $$p\left(x_{k}|Z_{k}\right)=\frac{p\left(z_{k}|x_{k}\right)p\left(x_{k}|Z_{k-1}\right)}{p\left(z_{k}|Z_{k-1}\right)}.$$

The optimal Bayesian solution based on Equations (5) and (7) is conceptual because the analytic solution of both equations cannot be determined in most practical situations. Therefore, numerical approximations have to be used.

### 3.2. The Particle Filter

In general, it is not possible to write a closed-form expression for the posterior pdf $p(x_{k}|Z_{k})$ in the nonlinear non-Gaussian case. Therefore, an approximate solution is required. The solution used in this study is referred to as the particle filter, which is a numerical approximation based on random sampling. Particle filters are applications of Monte Carlo methods to Bayesian estimation [56]. Background information on particle filtering can be found in, e.g., References [57,58,59,60]. The fundamental concept in the particle filter is to approximate the posterior pdf $p(x_{k}|Z_{k})$ as a weighted combination of sample points, also known as particles.

(8) $$p(x_{k}|Z_{k})\approx \sum_{p=1}^{P}w_{k}^{p}\delta \left(x_{k}-x_{k}^{p}\right),$$

where δ(x) is the Dirac delta function, $w_{k}^{p}$ refers to weights and sums to unity, and $x_{k}^{p}$ refers to particles. The convergence properties of the approximation were well studied [61,62]. The expectation of any nonlinear function of the state given the approximate posterior pdf can be evaluated as follows:

(9) $$E\left[g\left(x_{k}\right)|Z_{k}\right]\equiv \int g\left(x_{k}\right)p(x_{k}|Z_{k})dx_{k}\approx \sum_{p=1}^{P}w_{k}^{p}g\left(x_{k}^{p}\right).$$

The approximation of Equation (9) is referred to as Monte Carlo integration and can be sequentially applied to both Equations (5) and (7), i.e., the Chapman–Kolmogorov prediction and Bayesian update, respectively. Therefore, the particle filter, and Bayesian filtering in general, is also referred to as sequential Monte Carlo (SMC).

The particle filter algorithm, thus, provides a mechanism to recursively generate a set of weighted particles approximating $p(x_{k}|Z_{k})$ starting from a previous set of weighted particles approximating $p(x_{k-1}|Z_{k-1})$ in two steps (or phases): prediction and update. In the first phase, the particles are moved to new locations using a tractable pdf referred to as the proposal distribution, approximating the pdf of interest. In the second phase, new particle weights are determined to correct for the difference between the proposal distribution and the true pdf. This procedure is referred to as importance sampling [57].

The proposal distribution is a function selected by the system designer to cover the whole state space where the true distribution is non-zero. A poor selection of the proposal distribution lowers the efficiency of particle filters since many particles will be assigned very low weights and, therefore, a larger number of particles will be required to satisfy performance requirements. The sample–importance–resample (SIR), also denoted sequential importance resampling, particle filter is a common and popular implementation that uses the system dynamics as a proposal distribution, which is relatively straightforward and simplifies weight updating. In this study, a form of SIR particle filter was implemented, in which, for each particle $x_{k-1}^{p}$, a new particle $x_{k}^{p}$ is drawn from the transition pdf $p(x_{k}|x_{k-1}^{p})\;$, and then weights are updated by scaling the previous weights by the current measurement likelihood and renormalizing.

(10) $$w_{k}^{p}={\left(W_{k}\right)}^{-1}p\left(z_{k}|x_{k}^{p}\right)w_{k-1}^{p},$$

where $W_{k}=\sum_{p=1}^{P}p\left(z_{k}|x_{k}^{p}\right)w_{k-1}^{p}$ is the normalizing factor.

### 3.3. Particle Filter Implementation

Particle filtering was also implemented for positioning problems in, e.g., References [58,60,63,64,65]. In the SIR version of the particle filter, also known as a bootstrap filter or condensation algorithm, the particles are randomly generated from the motion (or dynamics) model. Sampling from the dynamics can be a very diffuse distribution. Thus, the proportion of particles that sample a trajectory close to the measurements may be very small. Therefore, a large number of particles may be required to represent the high-probability regions of the state space. Resampling is a strategy to improve the number of particles following trajectories with high likelihood. At each time step, copies of highly likely particles replace unlikely particles via a random sampling process. Some of the resampling strategies include multinomial resampling, stratified resampling, systematic resampling, and residual resampling [66,67].

The implemented particle filter is listed in Algorithm 1. Initially, there is no information about the position of the target node. Therefore, the particle filter generates P particles uniformly distributed in the whole working space. When the set of the three TDoA measurements, made by four anchor nodes, is available at the first time index, i.e., k=1, the weight $w_{k}^{p}$ of each particle $x_{k}^{p}$ is then computed as follows:

(11) $$w_{k}^{p}=\frac{1}{\sum_{i=1}^{3}{\left(dd_{k,i}-dd_{i}^{p}\right)}^{2}},$$

where $dd_{k,i}$ is the *i*-th TDoA measurement, i.e., range or distance difference measurement, at time $t_{k}$, i=1,…, 3, and $dd_{i}^{p}$ is the *i*-th distance difference of the particle $x_{k}^{p}$, which is the measurement model. The weight of each particle is inversely proportional to the similarity metric used, which is the sum of squared distances between $dd_{k,i}$ and $dd_{i}^{p}$.

The distance difference measurement $dd_{k,i}$ is expressed as

(12) $$dd_{k,i}=∥a_{i+1}-x_{k}∥-∥a_{1}-x_{k}∥+w_{i+1,1},\;i=1,\ldots ,\;3,\;\;$$

where $a_{i+1}$ is the known 3D position of the non-master anchor nodes in a 3D Cartesian coordinate system, $x_{k}$ is the estimated 3D position of the target node at time $t_{k}$, $a_{1}$ is the known 3D position of the reference or master anchor node, and $w_{i+1,1}$ is the measurement noise. The *i*-th distance difference of the particle $x_{k}^{p}$, i.e., the measurement model is computed as follows:

(13) $$dd_{i}^{p}=∥a_{i+1}-x_{k}^{p}∥-∥a_{1}-x_{k}^{p}∥,\;i=1,\ldots ,\;3.\;\;$$

A number L of the best-weighted particles, where L<P  , is selected, and the weights of these selected particles are normalized. The position $x_{k}$ of the target node is then estimated using these L selected particles and their normalized weights as follows:

(14) $$x_{k}=\frac{\sum_{p=1}^{L}x_{k}^{p}w_{k}^{p}}{\sum_{p=1}^{L}w_{k}^{p}},$$

which is the weighted trimmed average estimate (WTAE). The initialization phase is completed after computing the first estimate of the target node’s position, i.e., $x_{1}$.

In the prediction step, the particles are placed in new locations to account for the dynamics of the target node. A resampling procedure is used to generate uniformly distributed particles.

(15) $$x_{k}^{p}\sim U\left(x_{k-1}-R,\;x_{k-1}+R\right),\;k>1,$$

where $R=\left[\begin{array}{ccc} R_{x} & R_{y} & R_{z} \end{array}\right]$ is called the resampling space, where the resampling ranges $R_{x}$, $R_{y}$, and $R_{z}$, in the *x*-, *y*-, and *z*-directions, respectively, are assumed to be equal, i.e., $R_{x}=R_{y}=R_{z}=R$. Thus, the proposal distribution approximating the posterior pdf generates new particles, which are uniformly distributed within a cube of 2R side length and center at the previous state estimate $x_{k-1}$. When the value of R is properly determined, i.e., to consider the maximum expected displacement in the *x*-, *y*-, and *z*-directions during the measurement sampling time, the cloud of particles is continuously updated to ensure a good representation of the posterior pdf and acceptable accuracy of the particle filter while avoiding degeneracy over time. Sudden or unexpected movements can cause incorrect predictions that lower the diversity of the particle cloud and fail the particle filter. Therefore, the value of R should be selected to also account for such extreme situations to quickly converge to the true location or to recover from false locations. If convergence or recovery fails, the particle filter becomes stranded and has to be restarted using the last reliable measurements [68].

When new TDoA measurements are available at the next time index, the weights of the new particles are calculated using Equation (11), and then a new state estimate, i.e., new target node’s position, is computed using Equation (14). The iteration of the particle filter is further continued as indicated in Algorithm 1.
**Algorithm 1** The particle filter algorithm.**0. Initialization:** Generate P particles uniformly distributed in the whole working space. Compute the weight of each particle $x_{k}^{p}$ according to Equation (11), when TDoA measurements are available at time $t_{k}$,  k=1. Estimate the position of the target node, $x_{k}$, according to Equation (14). **1. Prediction:**
Generate new particles according to Equation (15). **2. Update:**
Compute the weight of each particle $x_{k}^{p}$ according to Equation (11), when TDoA measurements are available at time $t_{k}$,  k>1. **3. State Estimation:**
Estimate the position of the target node, $x_{k}$, according to Equation (14). Set k=k+1 and repeat from step 1.

## 4. Simulation Results

The performance of the proposed particle filter was evaluated by computer simulations using MATLAB. The root-mean-square error (RMSE) of the positioning solutions was investigated upon varying resampling range, R, values, and SNR levels. The TDoA, i.e., distance difference, measurements were simulated as true value plus a zero-mean Gaussian noise. The SNR of any measured TDoA signal is defined as

(16) $$SNR=10log\frac{dd_{k,i}^{2}}{\sigma_{i}^{2}}\;\left[\mathrm{dB}\right].$$

The variance of the Gaussian noise, $\sigma_{i}^{2}$, is then computed as

(17) $$\sigma_{i}^{2}=\frac{dd_{k,i}^{2}}{10^{SNR/10}}.$$

Two simulation environments were considered: (1) a small-size indoor environment to resemble applications such as an UAV indoor light show, which is a key performance at many ceremonies [45]; (2) a large-size indoor environment to resemble a UAV-based inventory management application. In the small-size environment, four simulation scenarios were considered with identical anchor node arrangement. The RMSE results were obtained via 100 simulation runs and are presented in Section 4.1, Section 4.2, Section 4.3 and Section 4.4. The RMSE results, in the large-size environment, were obtained via 50 simulation runs and are presented in Section 4.5. The implemented particle filter used a constant number of 1000 particles in all experiments. The position of the target node (UAV or drone) was estimated by Equation (14) using the 10% best-weighted particles, i.e., L=0.1×P.

### 4.1. Three-Dimensional Linear Path

**Figure 1:**
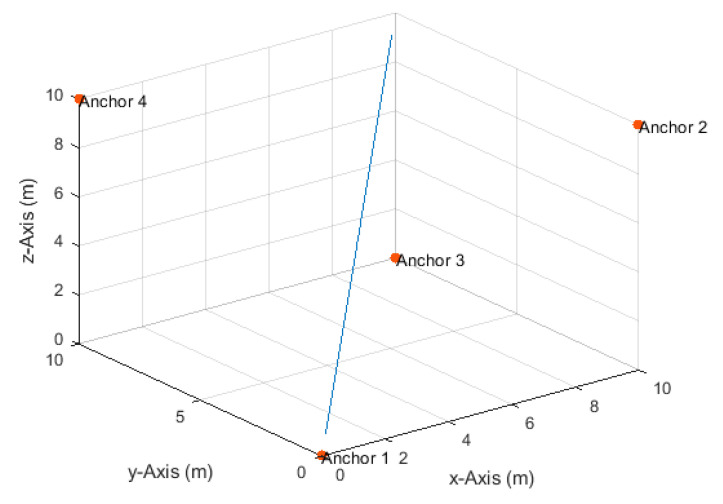
Anchor node arrangement and the three-dimensional (3D) linear path.

Four anchor nodes were placed at (0,0,0), (10,0,10), (10,10,0), and (0,10,10), and a drone moved along a linear path from position (0.5,0.5,0.5) to position (9.5,9.5,9.5) (see Figure 1) at constant velocities of 0.1 m/s in the *x*-, *y*-, and *z*-directions. Table 1 lists the total number of TDoA measurements obtained along the path and the traveled distances in the *x*-, *y*-, and *z*-directions between any two measurement times at all investigated measurement update rates. The maximum true distance difference, TDoA, value along the path was 12.58 m. Thus, the corresponding maximum standard deviation of the measurement errors according to Equation (17) at the investigated SNR levels of 20, 25, 30, 35, and 40 dB were, respectively, 126, 71, 40, 22, and 13 cm.

**Figure 2:**
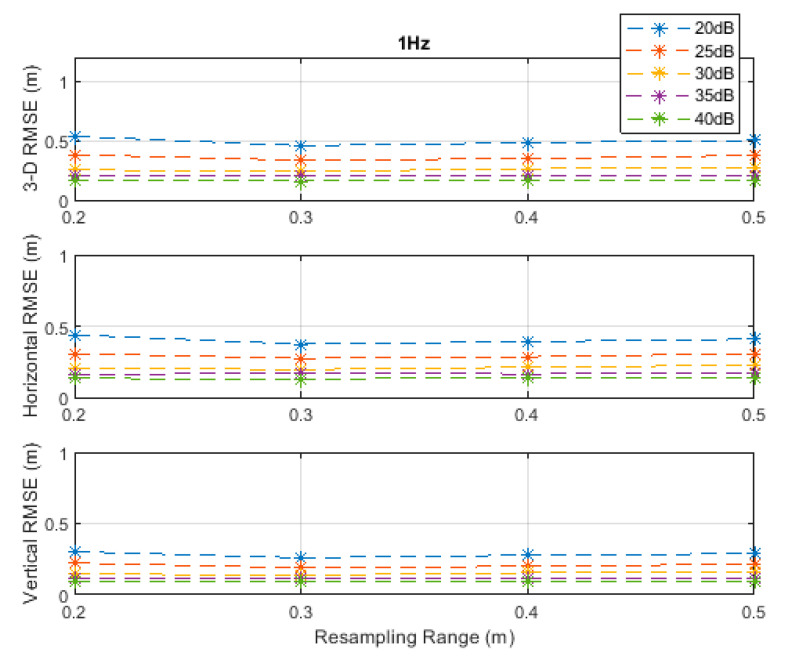
Root-mean-square error (RMSE) results along the 3D linear path at a measurement update rate of 1 Hz.

**Figure 3:**
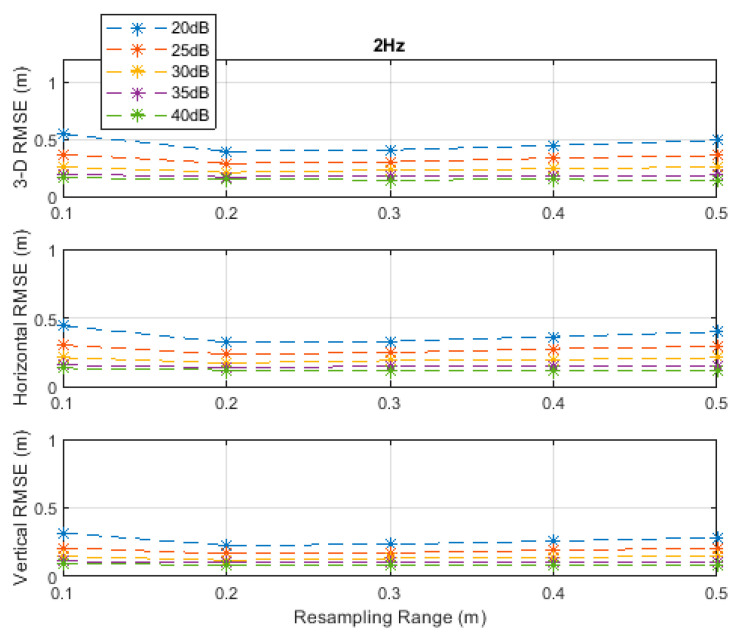
RMSE results along the 3D linear path at a measurement update rate of 2 Hz.

**Figure 4:**
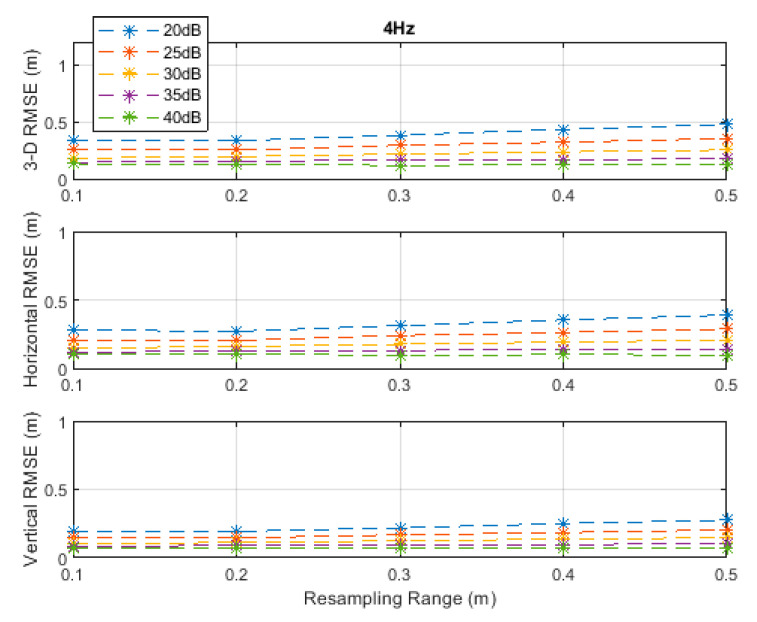
RMSE results along the 3D linear path at a measurement update rate of 4 Hz.

**Figure 5:**
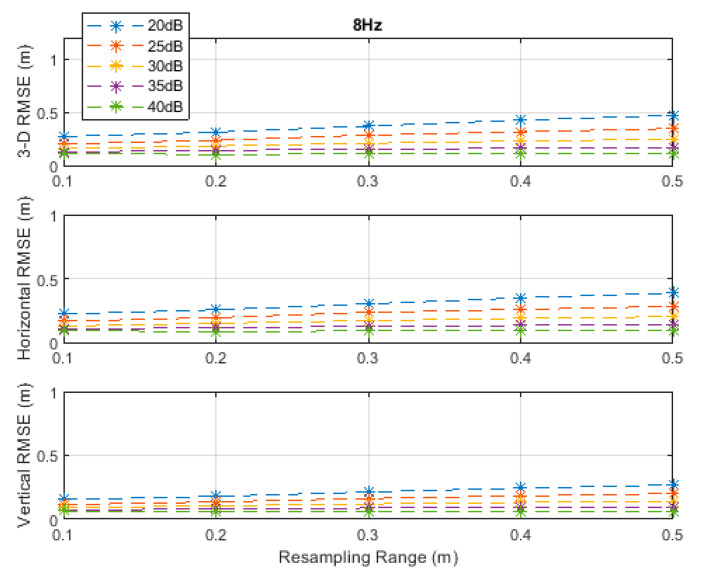
RMSE results along the 3D linear path at a measurement update rate of 8 Hz.

**Figure 6:**
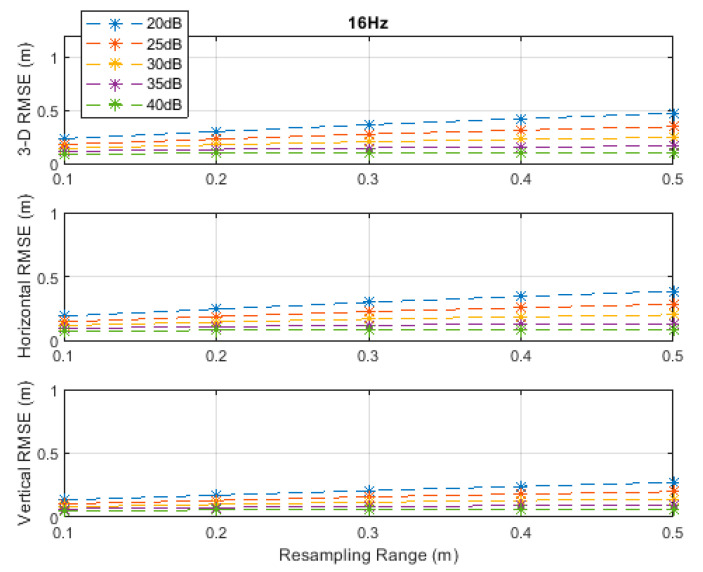
RMSE results along the 3D linear path at a measurement update rate of 16 Hz.

The RMSE results at the investigated measurement update rates are depicted in Figure 2, Figure 3, Figure 4, Figure 5 and Figure 6. The particle filter did not converge at an update rate of 1 Hz with a resampling range, R, of 0.1 m, over the investigated SNR levels, even if the initial position was known because the search volume was too small to allow the efficient working of the filtering mechanism. Therefore, the results with 0.1 m resampling range were omitted in Figure 2. The resampling range should be at least slightly greater than the traveled distance in the time between any two successive measurements, which was 0.1 m in the *x*-, *y*-, and *z*-directions at the measurement update rate of 1 Hz, to allow the particle filter to find and consider the correct volume containing the most likely drone positions, where the particle cloud should be placed.

It can be seen, from Figure 2, Figure 3, Figure 4, Figure 5 and Figure 6, that the positioning accuracy, in the RMSE sense, increased with increasing measurement update rate and SNR levels. Best accuracies can generally be obtained with a smaller resampling range if the measurement update rate and the SNR level are high enough. Thus, the search volume would be sufficient to account for measurement errors and the drone movements in the *x*-, *y*-, and *z*-directions. The obtained accuracy at the SNR level of 40 dB was almost constant over the resampling ranges 0.1 to 0.5 m at all investigated measurement update rates, since the traveled distance by the drone in the *x*-, *y*-, and *z*-directions was correctly contained within the search volumes suggested by the resampling ranges, and the measurement errors were small.

The standard deviations of the measurement errors at the SNR levels of 25 and 30 dB, i.e., 71 and 40 cm, respectively, seem to be feasible for real-world scenarios with dimensions similar to the simulated scenario. The horizontal and vertical RMSEs at the SNR level of 25 dB were about 14 and 10 cm, and those at the SNR level of 30 dB were about 12 and 8 cm, respectively, at a measurement update rate of 16 Hz. The best accuracies (from Figure 2, Figure 3, Figure 4, Figure 5 and Figure 6), rounded to two significant digits, obtained at all investigated SNR levels and measurement update rates are summarized in Table 2. The accuracies increased from left to right as the SNR level increased, and from top to bottom as the measurement update rate increased. Since the SNR levels of 25 and 30 dB, corresponding to measurement errors of 71 and 40 cm, respectively, can be achieved in a similar real-world scenario, a horizontal accuracy of 20 cm or less and a vertical accuracy of 15 cm or less could be obtained if at least a measurement update rate of 4 Hz is implemented, as can be seen from Table 2.

### 4.2. Horizontal Linear Path

**Figure 7:**
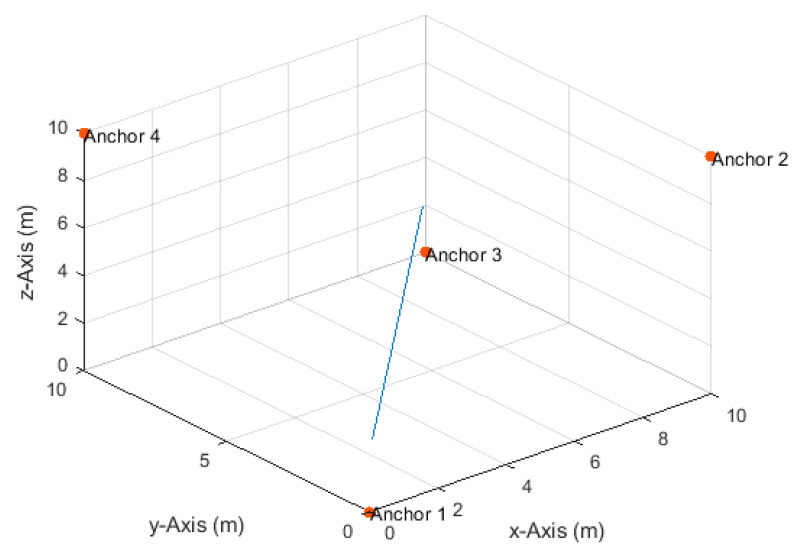
Anchor node arrangement and the horizontal linear path.

The drone moved along a horizontal linear path from position (0.5,0.5,2.5) to the position (9.5,9.5,2.5) (Figure 7), at a constant height of 2.5 m and constant velocities of 0.1 m/s in the *x*- and *y*-directions. The total number of measurements and traveled distances, in the *x*- and *y*-directions only, along the path at the investigated measurement update rates were identical to the values listed in Table 1. The maximum true distance difference along the path was 11.07 m. Thus, the corresponding maximum standard deviation of the measurement errors according to Equation (17) at the investigated SNR levels of 20, 25, 30, 35, and 40 dB were, respectively, 111, 62, 35, 20, and 11 cm.

The positioning accuracy behavior in the RMSE sense, at all SNR levels, measurement update rates, and resampling ranges, was similar to the 3D linear path case investigated in the previous subsection. Therefore, the plots of the results are skipped for brevity, and only the summary of these results is listed in Table 3. A horizontal accuracy of 22 cm or less and a vertical accuracy of 15 cm or less could be obtained at an SNR of 25 dB or higher if at least a measurement update rate of 4 Hz is achieved, as can be seen from Table 3.

### 4.3. Horizontal Circular Path

**Figure 8:**
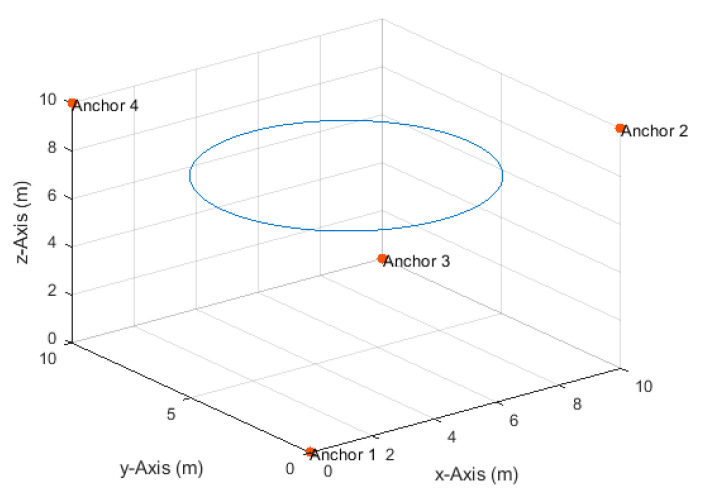
Anchor node arrangement and the horizontal circular path.

The drone moved on a horizontal circular path, with a radius of 4 m and center at (5,5,7.5) (Figure 8), at a constant height of 7.5 m and a constant angular velocity of $\frac{\pi }{50}$ rad/s. The drone completed a single full round starting from position (9,5,7.5). The corresponding velocities in the *x*- and *y*-directions varied between a minimum of 0.008 m/s and a maximum of 0.25 m/s. The total number of TDoA measurements obtained along the path at the measurement update rates of 1, 2, 4, 8, and 16 Hz were, respectively, 101, 201, 401, 801, and 1601. The maximum true distance difference along the path was 7.53 m. Thus, the corresponding maximum standard deviation of the measurement errors according to Equation (17) at the investigated SNR levels of 20, 25, 30, 35, and 40 dB were, respectively, 75, 42, 24, 13, and 8 cm.

**Figure 9:**
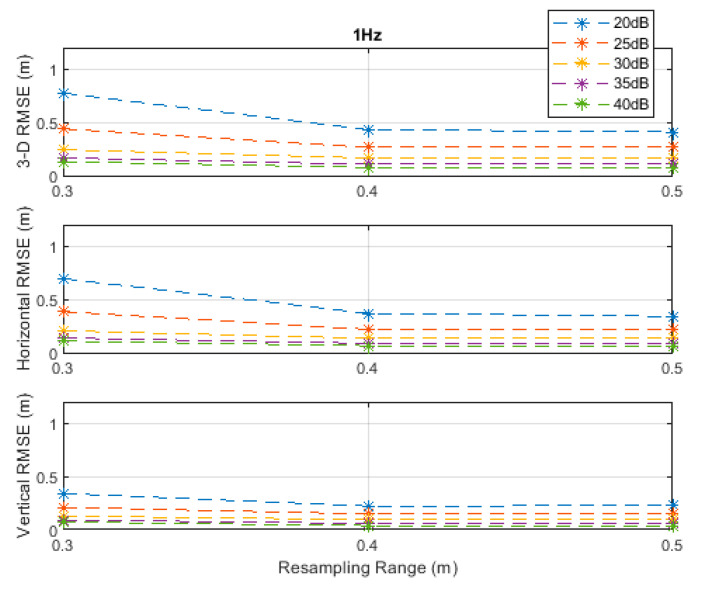
RMSE results along the horizontal circular path at a measurement update rate of 1 Hz.

**Figure 10:**
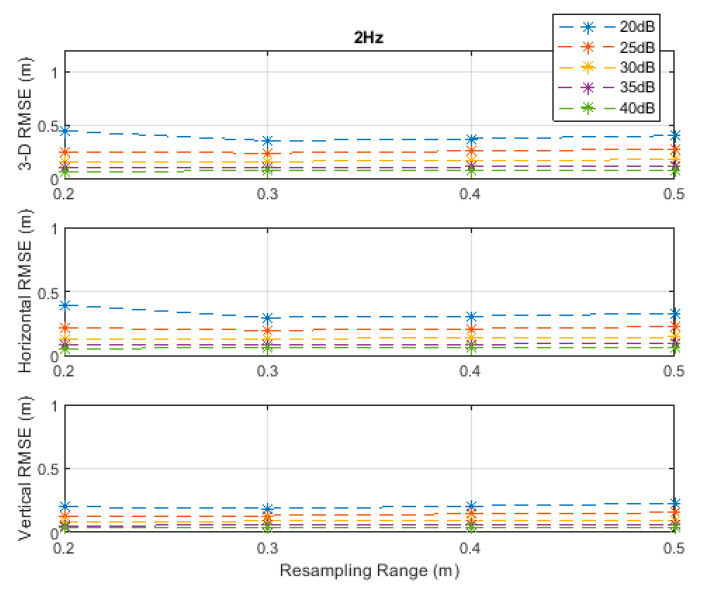
RMSE results along the horizontal circular path at a measurement update rate of 2 Hz.

**Figure 11:**
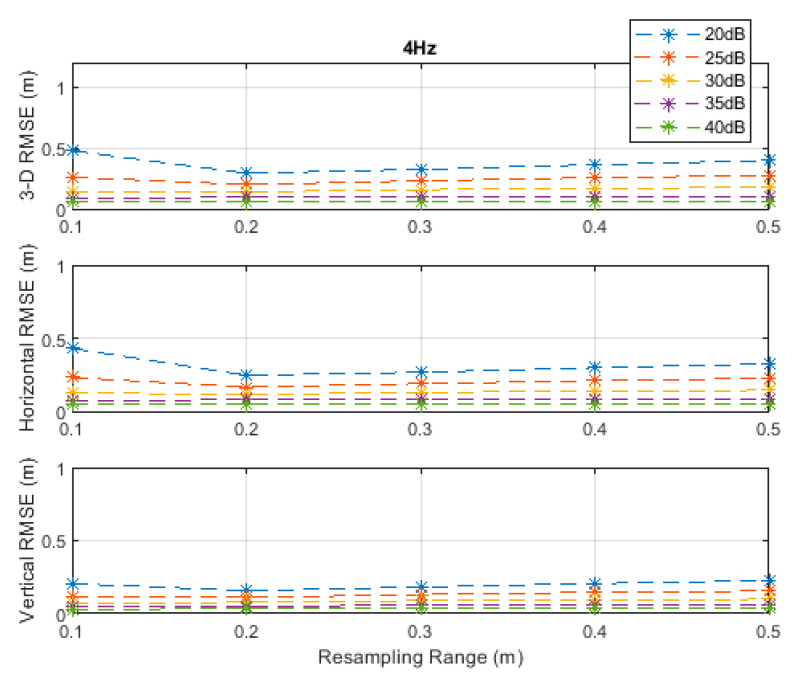
RMSE results along the horizontal circular path at a measurement update rate of 4 Hz.

**Figure 12:**
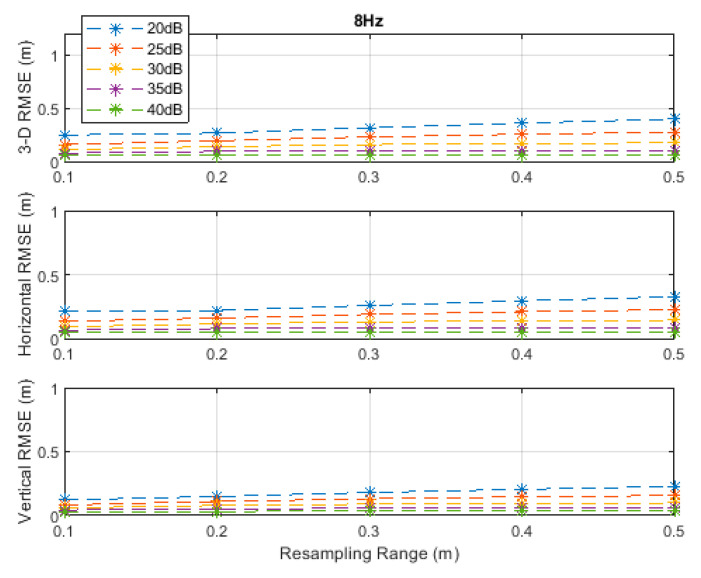
RMSE results along the horizontal circular path at a measurement update rate of 8 Hz.

**Figure 13:**
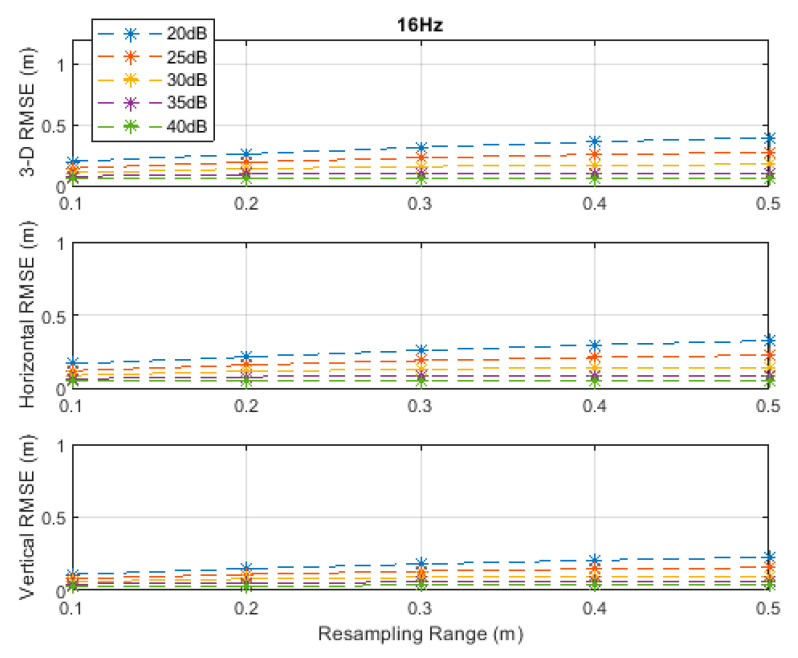
RMSE results along the horizontal circular path at a measurement update rate of 16 Hz.

At a measurement update rate of 1 Hz, the particle filter did not converge with the resampling ranges of 0.1 and 0.2 m, regardless of the SNR level, since both ranges were less than the maximum distance of 0.25 m that could be traveled in the *x*- and *y*-directions between any two measurement times. Thus, the particle filter would not find the correct volume of likely drone positions. Therefore, the results with the resampling ranges of 0.1 and 0.2 m are omitted in Figure 9. The particle filter did not also converge at a measurement update rate of 2 Hz with the resampling range of 0.1 m since the maximum distance that could be traveled in the *x*- and *y*-directions between any two measurement times was equal to 0.125 m. Therefore, the results with a resampling range of 0.1 m are also omitted in Figure 10. RMSE results at the measurement update rates of 4, 8, and 16 Hz are shown in Figure 11, Figure 12 and Figure 13, respectively.

The best accuracies, rounded to two significant digits, obtained at all investigated SNR levels and measurement update rates are summarized in Table 4. A horizontal accuracy of 16 cm or less and a vertical accuracy of 11 cm or less could be obtained at an SNR of 25 dB or higher if at least a measurement update rate of 4 Hz is achieved, as can be seen from Table 4.

### 4.4. Helical Path

**Figure 14:**
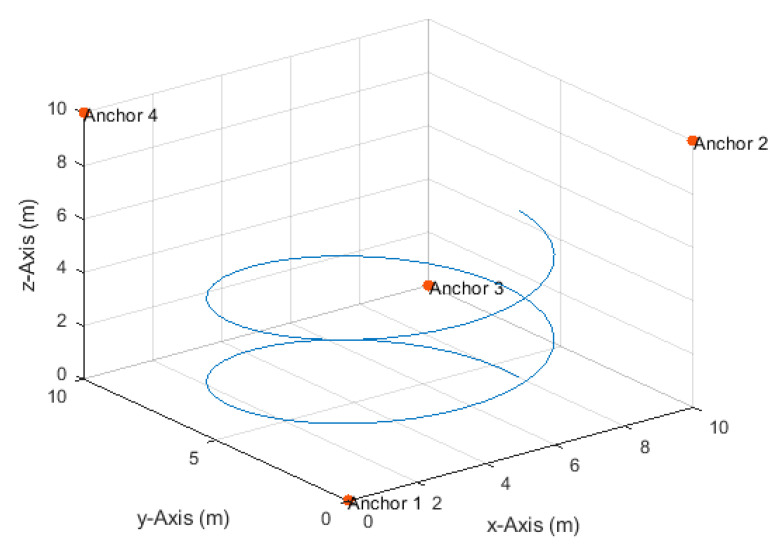
Anchor node arrangement and the helical path.

The drone moved on a helical path, with a radius of 4 m (Figure 14), at a constant horizontal angular velocity of $\frac{\pi }{20}$ rad/s and a constant vertical velocity of 7.85 cm/s from position (8,4,0) to position (8,4,6.28). The corresponding velocities in the *x*- and *y*-directions varied between a minimum of 0.05 m/s and a maximum of 0.63 m/s.

The total number of TDoA measurements obtained along the path at the measurement update rates of 4, 8, and 16 Hz were, respectively, 321, 641, and 1281. The maximum true distance difference along the path was 10.07 m. Thus, the corresponding maximum standard deviation of the measurement errors according to Equation (17) at the investigated SNR levels of 20, 25, 30, 35, and 40 dB were, respectively, 101, 57, 32, 18, and 10 cm.

**Figure 15:**
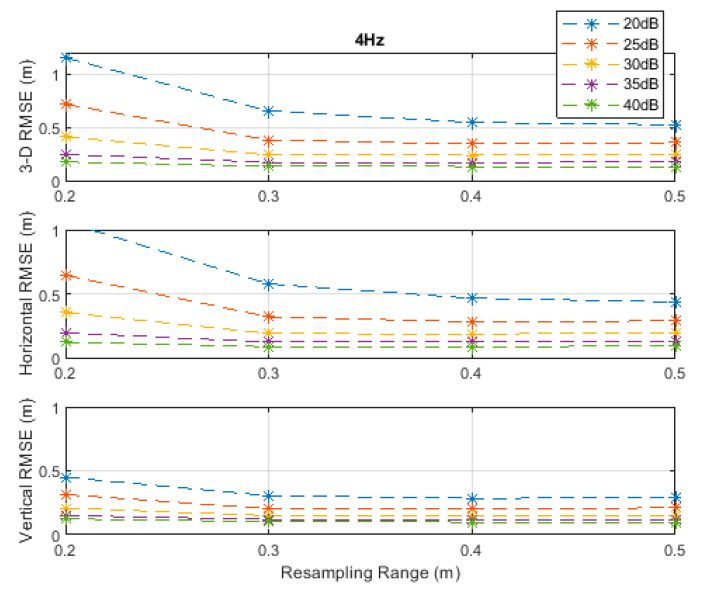
RMSE results along the helical path at a measurement update rate of 4 Hz.

**Figure 16:**
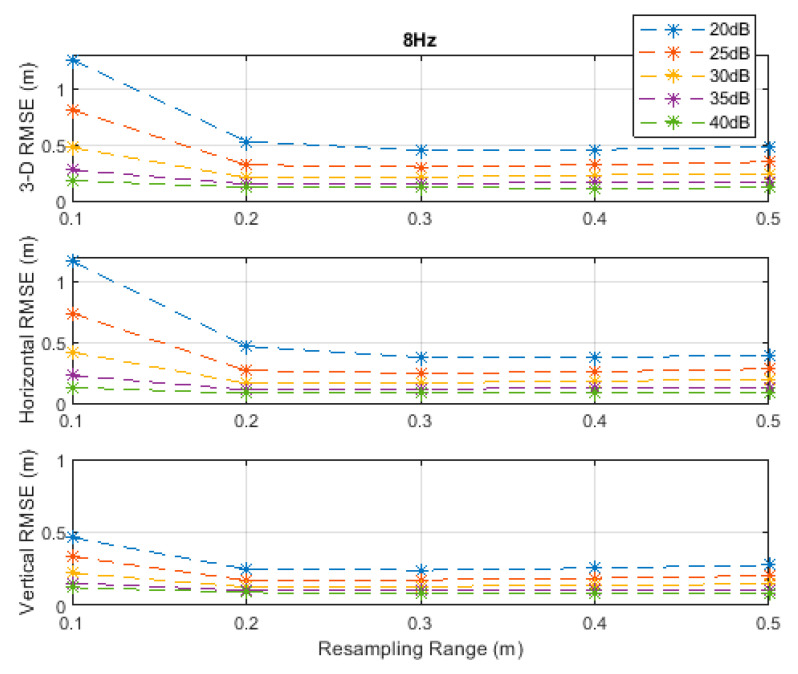
RMSE results along the helical path at a measurement update rate of 8 Hz.

**Figure 17:**
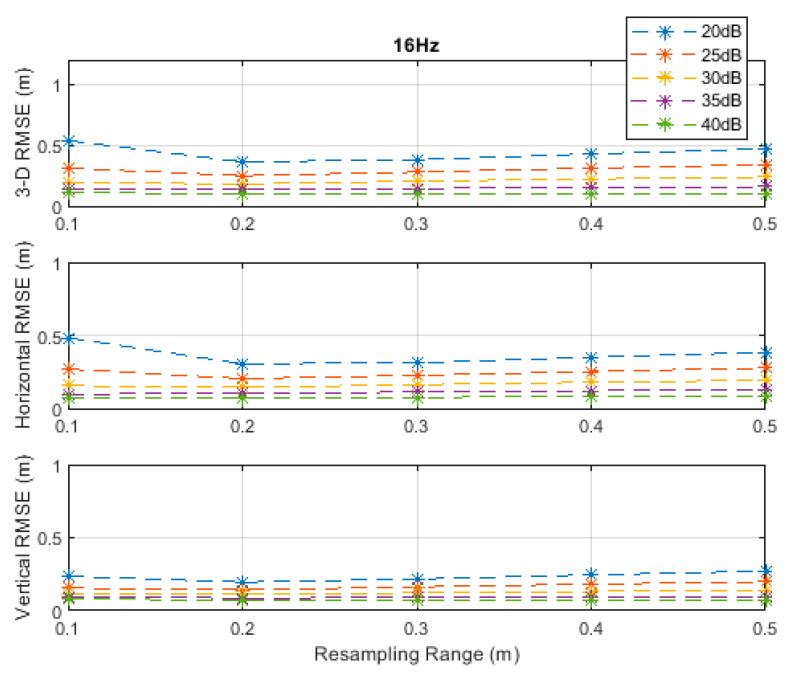
RMSE results along the helical path at a measurement update rate of 16 Hz.

Positioning accuracy results at measurement update rates of 4, 8, and 16 Hz are illustrated in Figure 15, Figure 16 and Figure 17, respectively, and summarized in Table 5. In this higher-dynamics scenario, an SNR level of at least 30 dB was required to obtain a horizontal accuracy below 20 cm at the 4 and 8 Hz measurement update rates. The obtained vertical accuracy was 15 cm or less. At a measurement update rate of 16 Hz, horizontal and vertical accuracies of 20 and 14 cm, respectively, could already be obtained at an SNR level of 25 dB.

### 4.5. An Inventory Management Scenario

In Reference [69], a four-anchor UWB system ranging up to 125 m at an update rate of 40 Hz was deployed. UWB two-way ranging at 80 Hz was reported in Reference [45]. Six anchors, placed on the ground and the ceiling, in addition to IMU measurements, were used for 3D localization. A range measurement accuracy of about 10 cm was achieved over a working range of a few hundred meters. The ranging accuracy had the same level irrespective of the target–anchor distance. UWB two-way ranging schemes were designed in Reference [47] and reached minimum and maximum practical measurement update rates of 62 and 372 Hz, respectively. The positioning system was applied to a 400-m circular track covered by 20 anchors, where reliable ranging up to 80 m and occasionally up to 220 m was obtained.

**Figure 18:**
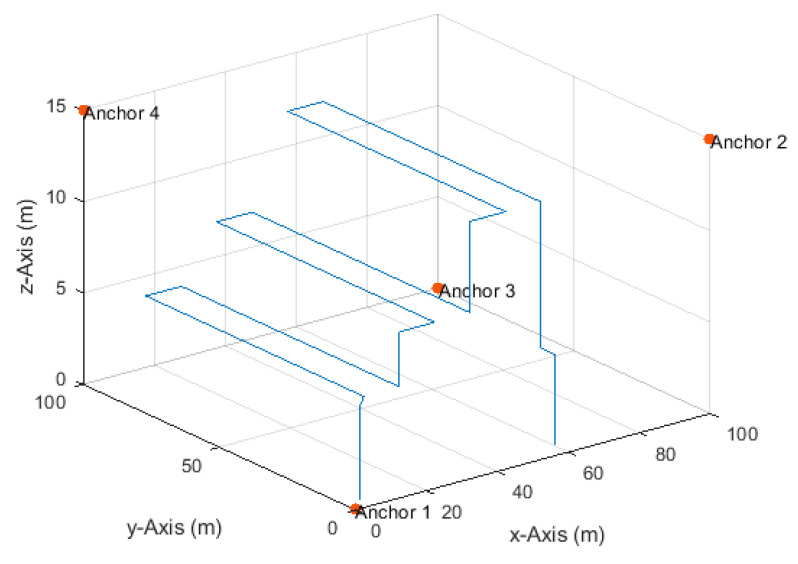
Anchor node arrangement and the linear paths.

The settings of this simulation resemble a realistic UAV-based inventory management scenario. Four anchor nodes were placed at (0,0,0), (100,0,15), (100,100,0), and (0,100,15), and a drone moved along several linear paths from position (5,5,0) to position (60,5,0) (Figure 18), at constant velocities of 0.5 m/s in the corresponding direction. TDoA measurements were obtained at a rate of 20 Hz with a total number of 22,658 measurements along the paths. Thus, the traveled distance between any two measurements was 2.5 cm in the corresponding direction. The maximum true distance difference value along the paths was 127.3 m. Therefore, the SNR level was set to 50 dB to yield a maximum standard deviation of the measurement errors of about 40 cm according to Equation (17).

**Figure 19:**
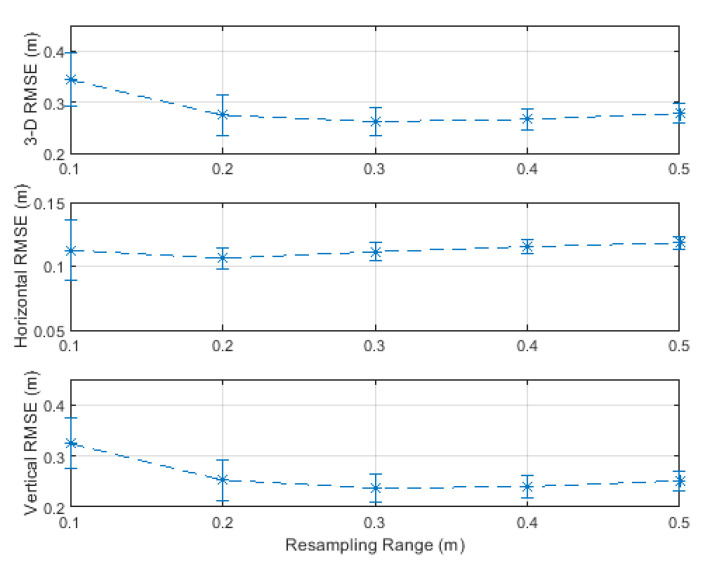
RMSE results of the unmanned aerial vehicle (UAV)-based inventory management scenario and corresponding error bars at a signal-to-noise ratio (SNR) of 50 dB and measurement update rate of 20 Hz.

The positioning accuracy was investigated with varying resampling ranges from 0.1 to 0.5 m. The RMSE results are plotted with error bars, representing the standard deviation, in Figure 19, and are listed in Table 6. It can be seen from Table 6 that a resampling range, R, of 0.3 m delivered the best combination of horizontal and vertical accuracies of 11 and 24 cm, respectively. However, the horizontal accuracy was similar (11–12 cm) over the investigated resampling ranges, and the vertical accuracy was also similar (24–25 cm) over resampling ranges greater than 0.1 m. The error bars in Figure 19 (their values are listed in Table 6) show that the vertical errors fluctuated higher than the horizontal errors.

**Figure 20:**
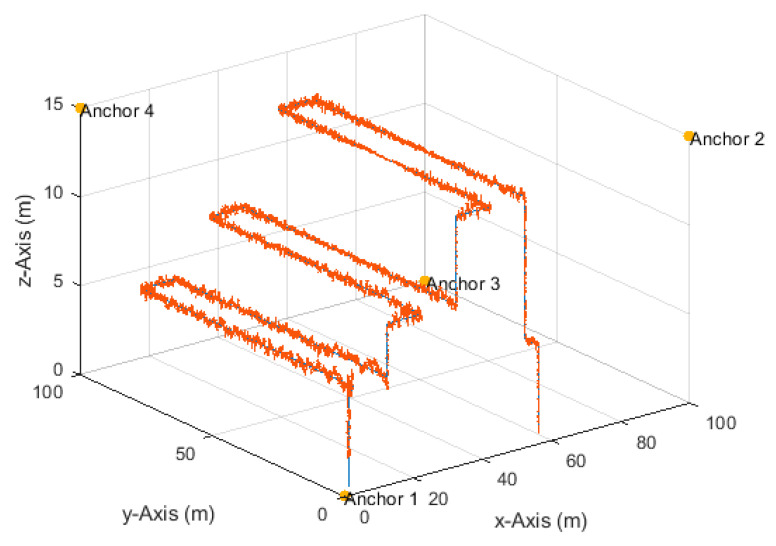
The particle filter position estimates (red) and ground truth (blue) of a single simulation run of the UAV-based inventory management scenario with a sampling range, R, of 0.3 m, and at an SNR of 50 dB and measurement update rate of 20 Hz.

Figure 20 shows the particle filter position estimates of a single simulation run with a resampling range, R, of 0.3 m. It can be seen that the particle filter required more time to converge to the correct vertical position, as illustrated in the initial phase. The particle filter also needed time to recover after sudden direction changes. It can also be noted that the vertical position estimates were more accurate and smoother during vertical movements and fluctuated during the horizontal movements. This fluctuation was due to the applied resampling range that dictated considering vertical position candidates above and below the true horizontal path. Therefore, fusing height information from, e.g., a barometer, can refine the value of the resampling range in the vertical dimension and, thus, smoother vertical position estimates can be obtained during horizontal movements. The 3D, horizontal, and vertical RMSEs, rounded to two significant digits, of the simulation run depicted in Figure 20 were 26, 11, and 24 cm, respectively, i.e., identical to the results over 50 simulation runs listed in Table 6 (column with resampling range of 0.3 m).

To avoid the bias in the RMSE results of Figure 19, it is common to discard 2% of the position estimates with the highest errors due to the convergence and recovery phases [70,71]. The 3D, horizontal, and vertical RMSEs after discarding 1% and 2% of the position estimates with the highest errors were, respectively, 20, 10, and 17 cm, and 19, 10, and 16 cm. Theses accuracies are acceptable for inventory management applications and indicate that the proposed particle filter works well under the assumed measurement update rate and error conditions.

The mean computation time required for a single run of the filter with 1000 particles, $T_{mean}^{1000}$, was estimated on two platforms: (1) an Intel Core i5 central processing unit (CPU) at 2.67 GHz with 8 GB random-access memory (RAM) running MATLAB R2018b, and (2) an Intel Core i7 CPU at 2.2 GHz with 16 GB RAM running MATLAB R2019b. The estimated $T_{mean}^{1000}$ values on both platforms were about 20 and 14 ms, i.e., allowing the proposed particle filter to work at about 50 and 70 Hz, respectively, within the MATLAB environment.

## 5. Conclusions

The TDoA measurement technique is widely used for position estimation. TDoA-based positioning is a nonlinear estimation problem. A particle filter algorithm was proposed to solve the four-anchor TDoA-based 3D positioning problem. The proposed particle filter was demonstrated via simulation studies for enabling UAV indoor positioning applications. It works with the minimum number of anchor nodes, from the mathematical point of view, required for 3D positioning and, thus, increases the availability of positioning systems, usually employs more than four anchors, in the case of anchor node failures and bad geometric dilution of precision (GDOP) conditions, and reduces the overall system hardware costs. The assumed working conditions can be achieved by UWB wireless technology. Therefore, it is possible to enable UAV positioning in, e.g., large warehouses for inventory management applications without the need for an excessive number of anchor nodes.

The correct arrangement and location calibration of the anchor nodes in the 3D space are important design criteria to get accurate 3D position estimates. Therefore, the arrangement of the anchor nodes has to be optimized to meet the accuracy requirements of the target application.

The simple implementation with any kind of model and noise assumption is the main reason for the wide application of particle filters. If the proposed resampling range, used in the prediction step of the particle filter, is correctly set, it can help to obtain better state predictions than directly integrating acceleration information from an IMU [45]. The particle filter also enables easy incorporation of further useful information, e.g., map data, and plain integration of further sensors, e.g., IMU, barometer, cameras, etc.

UAV indoor applications are increasing rapidly due to the variety of sensors that can be available onboard. Therefore, hybrid and complementary positioning systems should be used with sufficient computing power to tackle difficulties such as velocity estimation, signal multipath effects, and non-line-of-sight (NLoS) propagation errors. Candidate systems include IMUs, cameras, ultrasound devices, and signals of opportunity. Thus, with a proper regulatory framework for UAV usage [19] and more advanced UAV technologies, an endless number of more complex applications and sophisticated use cases can be developed and implemented on top of the proposed approach. This implies future works toward integrating IMU and visual information from cameras and considering other practical sources of error such as multipath, NLoS propagation, anchor node clock synchronization, and optimal anchor node placement. Other interesting future work topics include (1) auto-calibration of the anchor nodes to determine their relative positions and reduce installation time in rapid deployment ad hoc applications, (2) formation flight of multi-UAV in a variety of indoor and outdoor environments, and (3) enabling cooperative positioning among UAVs.

## Figures and Tables

**Table 1:** The total number of measurements obtained along the three-dimensional (3D) linear path at all investigated measurement update rates, and the corresponding traveled distances.

Measurement Update Rate (Hz)	Total No. of Measurements	Traveled Distance (cm)
1	91	10
2	181	5
4	361	2.5
8	721	1.25
16	1441	0.625

**Table 2:** Best accuracies obtained at all investigated signal-to-noise ratio (SNR) levels and measurement update rates in the 3D linear path experiments.

RMSE (m)	20 dB	25 dB	30 dB	35 dB	40 dB	Measurement Update Rate
3D	0.46	0.33	0.24	0.20	0.16	
Horizontal	0.37	0.27	0.20	0.16	0.13	1 Hz
Vertical	0.26	0.19	0.14	0.12	0.09	
3D	0.39	0.29	0.21	0.17	0.14	
Horizontal	0.32	0.24	0.17	0.14	0.11	2 Hz
Vertical	0.23	0.16	0.12	0.10	0.08	
3D	0.33	0.25	0.18	0.14	0.12	
Horizontal	0.27	0.20	0.15	0.12	0.10	4 Hz
Vertical	0.19	0.15	0.10	0.08	0.07	
3D	0.27	0.20	0.16	0.13	0.10	
Horizontal	0.22	0.17	0.13	0.10	0.08	8 Hz
Vertical	0.16	0.11	0.09	0.07	0.06	
3D	0.23	0.18	0.14	0.11	0.09	
Horizontal	0.19	0.14	0.12	0.09	0.07	16 Hz
Vertical	0.13	0.10	0.08	0.06	0.05	

**Table 3:** Best accuracies obtained at all investigated SNR levels and measurement update rates in the horizontal linear path experiments.

RMSE (m)	20 dB	25 dB	30 dB	35 dB	40 dB	Measurement Update Rate
3D	0.48	0.34	0.26	0.20	0.16	
Horizontal	0.39	0.28	0.21	0.16	0.14	1 Hz
Vertical	0.28	0.20	0.14	0.11	0.08	
3D	0.41	0.31	0.23	0.18	0.13	
Horizontal	0.33	0.25	0.19	0.15	0.11	2 Hz
Vertical	0.24	0.18	0.13	0.09	0.07	
3D	0.36	0.26	0.19	0.15	0.11	
Horizontal	0.30	0.22	0.16	0.13	0.10	4 Hz
Vertical	0.21	0.15	0.11	0.08	0.06	
3D	0.30	0.22	0.17	0.14	0.10	
Horizontal	0.24	0.18	0.14	0.11	0.08	8 Hz
Vertical	0.18	0.13	0.10	0.08	0.05	
3D	0.26	0.19	0.14	0.12	0.09	
Horizontal	0.21	0.16	0.12	0.10	0.08	16 Hz
Vertical	0.15	0.11	0.08	0.07	0.05	

**Table 4:** Best accuracies obtained at all investigated SNR levels and measurement update rates in the horizontal circular path experiments.

RMSE (m)	20 dB	25 dB	30 dB	35 dB	40 dB	Measurement Update Rate
3D	0.41	0.26	0.17	0.11	0.08	
Horizontal	0.34	0.22	0.13	0.09	0.06	1 Hz
Vertical	0.23	0.15	0.10	0.06	0.04	
3D	0.35	0.24	0.15	0.10	0.06	
Horizontal	0.30	0.19	0.12	0.08	0.05	2 Hz
Vertical	0.19	0.13	0.08	0.05	0.03	
3D	0.29	0.20	0.14	0.09	0.06	
Horizontal	0.25	0.16	0.11	0.07	0.05	4 Hz
Vertical	0.16	0.11	0.07	0.05	0.03	
3D	0.25	0.16	0.11	0.08	0.06	
Horizontal	0.22	0.13	0.09	0.06	0.05	8 Hz
Vertical	0.12	0.08	0.06	0.04	0.03	
3D	0.20	0.14	0.10	0.08	0.05	
Horizontal	0.17	0.12	0.09	0.06	0.04	16 Hz
Vertical	0.11	0.08	0.06	0.04	0.03	

**Table 5:** Best accuracies obtained at all investigated SNR levels and measurement update rates in the helical path experiments.

RMSE (m)	20 dB	25 dB	30 dB	35 dB	40 dB	Measurement Update Rate
3D	0.52	0.34	0.24	0.17	0.13	
Horizontal	0.44	0.28	0.18	0.12	0.09	4 Hz
Vertical	0.28	0.20	0.15	0.12	0.10	
3D	0.45	0.30	0.20	0.15	0.11	
Horizontal	0.38	0.24	0.16	0.11	0.08	8 Hz
Vertical	0.24	0.17	0.13	0.10	0.08	
3D	0.36	0.25	0.18	0.13	0.10	
Horizontal	0.31	0.20	0.14	0.10	0.07	16 Hz
Vertical	0.20	0.14	0.11	0.09	0.07	

**Table 6:** RMSE results of the UAV-based inventory management scenario.

RMSE (m)	Resampling Range (m)				
	0.1	0.2	0.3	0.4	0.5
3D	0.34	0.27	0.26	0.27	0.28
*1-σ* (cm)	*5.2*	*3.9*	*2.6*	*2.1*	*1.9*
Horizontal	0.11	0.11	0.11	0.12	0.12
*1-σ* (cm)	*2.3*	*0.8*	*0.7*	*0.5*	*0.5*
Vertical	0.32	0.25	0.24	0.24	0.25
*1-σ* (cm)	*4.9*	*4.0*	*2.7*	*2.1*	*1.9*

## Abbreviations

2-D	Two-dimensional
3-D	Three-dimensional
AUV	Autonomous underwater vehicles
EKF	Extended Kalman filter
GDOP	Geometric dilution of precision
GNSS	Global navigation satellite system
IMU	Inertial measurement unit
INS	Inertial navigation system
IoV	Internet of vehicles
KF	Kalman filter
LBS	Location-based services
LoS	Line-of-sight
MAV	Micro aerial vehicle
MCL	Monte Carlo localization
NLoS	Non-line-of-sight
pdf	Probability density function
RF	Radiofrequency
RMSE	Root-mean-square error
SIR	Sample–importance–resample, sequential importance resampling
SMC	Sequential Monte Carlo
SNR	Signal-to-noise ratio
TDoA	Time difference of arrival
ToA	Time of arrival
ToF	Time of flight
UAV	Unmanned air vehicle
UKF	Unscented Kalman filter
UWB	Ultra-wide band
VLC	Visible light communications
WTAE	Weighted trimmed average estimate

## References

[1] Smith J.O., Abel J.S. (1987). Closed-form least-squares source location estimation from range-difference measurements. IEEE Trans. Acoust. Speech Signal Process..

[2] Chan Y.T., Ho K.C. (1994). Simple and efficient estimator for hyperbolic location. IEEE Trans. Signal Process..

[3] Dogancay K., Hashemi-Sakhtsari A. (2005). Target tracking by time difference of arrival using recursive smoothing. Signal Process..

[4] Xiao W., Weng Y., Xie L. (2011). Total least squares method for robust source localization in sensor networks using TDOA measurements. Int. J. Distrib. Sens. Netw..

[5] Lin L., So H.C., Chan F.K.W., Chan Y.T., Ho K.C. (2013). A new constrained weighted least squares algorithm for TDOA-based localization. Signal Process..

[6] Khalaf-Allah M. An extended closed-form least-squares solution for three-dimensional hyperbolic geolocation. Proceedings of the 2014 IEEE Symposium on Industrial Electronics Applications (ISIEA).

[7] Khalaf-Allah M. (2020). Performance Comparison of Closed-Form Least Squares Algorithms for Hyperbolic 3-D Positioning. J. Sens. Actuator Netw..

[8] Lui K.W., Chan F.K., So H.C. (2009). Semidefinite programming approach for range-difference based source localization. IEEE Trans. Signal Process..

[9] Li T., Ekpenyong A., Huang Y.-F. (2006). Source localization and tracking using distributed asynchronous sensors. IEEE Trans. Signal Process..

[10] Win M.Z., Conti A., Mazuelas S., Shen Y., Gifford W.M., Dardari D., Chiani M. (2011). Network localization and navigation via cooperation. IEEE Commun. Mag..

[11] Gholami M.R., Gezici S., Strom E.G. (2012). Improved position estimation using hybrid TW-TOA and TDOA in cooperative networks. IEEE Trans. Signal Process..

[12] Cakir O., Kaya I., Yazgan A. Propagation speed free emitter location finding using TDOA. Proceedings of the 21st Telecommunications Forum (TELFOR ’13).

[13] De Sanctis G., Rovetta D., Sarti A., Scarparo G., Tubaro S. Localization of tactile interactions through TDOA analysis: Geometric vs. inversion-based method. Proceedings of the 14th European Signal Processing Conference (EUSIPCO ’06).

[14] Chen J.C., Yao K., Hudson R.E. (2002). Source localization and beamforming. IEEE Signal Process. Mag..

[15] Priyantha N.B., Balakrishnan H., Demaine E.D., Teller S. Mobile-assisted localization in wireless sensor networks. Proceedings of the IEEE 24th Annual Joint Conference of the IEEE Computer and Communications Societies.

[16] Díez-González J., Álvarez R., Sánchez-González L., Fernández-Robles L., Pérez H., Castejón-Limas M. (2019). 3D Tdoa Problem Solution with Four Receiving Nodes. Sensors.

[17] Nonami K. (2007). Prospect and recent research & development for civil use autonomous unmanned aircraft as UAV and MAV. J. Syst. Design Dyn..

[18] Tomic T., Schmid K., Lutz P., Domel A., Kassecker M., Mair E., Grixa I.L., Ruess F., Suppa M., Burschka D. (2012). Toward a fully autonomous UAV: Research platform for indoor and outdoor urban search and rescue. IEEE Robot. Autom. Mag..

[19] López L.B., van Manen N., van der Zee E., Bos S., Nurmi J., Lohan E.-S., Wymeersch H., Seco-Granados G., Nykänen O. (2017). DroneAlert: Autonomous Drones for Emergency Response. Multi-Technology Positioning.

[20] Xu Y., Choi J., Dass S., Maiti T. (2017). Bayesian Prediction and Adaptive Sampling Algorithms for Mobile Sensor Networks.

[21] Sukhatme G.S., Dhariwal A., Zhang B., Oberg C., Stauffer B., Caron D.A. (2007). Design and development of a wireless robotic networked aquatic microbial observing system. Environ. Eng. Sci..

[22] Wang Y., Tan R., Xing G., Tan X., Wang J., Zhou R. (2014). Spatiotemporal aquatic field reconstruction using cyber-physical robotic sensor systems. ACM Trans. Sensor Netw. (TOSN).

[23] Laut J., Henry E., Nov O., Porfiri M. (2014). Development of a mechatronics-based citizen science platform for aquatic environmental monitoring. IEEE Trans. Mechatron..

[24] Choi J., Milutinovic D. (2015). Tips on stochastic optimal feedback control and Bayesian spatiotemporal models: Applications to robotics. J. Dyn. Syst. Meas. Control..

[25] Mupparapu S.S., Chappell S.G., Komerska R.J., Blidberg D.R., Nitzel R., Benton C., Popa D.O., Sanderson A.C. Autonomous systems monitoring and control (ASMAC)—An AUV fleet controller. Proceedings of the 2004 IEEE/OES Autonomous Underwater Vehicles.

[26] Nickell C.L., Woolsey C.A., Stilwell D.J. A low-speed control module for a stream lined AUV. Proceedings of the OCEANS 2005 MTS/IEEE.

[27] Bandyopadhyay P.R. (2005). Trends in biorobotic autonomous undersea vehicles. IEEE J. Ocean. Eng..

[28] Liu Y., Shen Y. UAV-Aided High-Accuracy Relative Localization of Ground Vehicles. Proceedings of the IEEE International Conference on Communications (ICC).

[29] Zeng Y., Zhang R., Lim T.J. (2016). Wireless communications with unmanned aerial vehicles: Opportunities and challenges. IEEE Commun. Mag..

[30] Matolak D.W., Sun R. (2015). Unmanned aircraft systems: Air-ground channel characterization for future applications. IEEE Veh. Technol. Mag..

[31] Mario G., Eun-Kyu L., Giovanni P., Uichin L. Internet of vehicles: From intelligent grid to autonomous cars and vehicular clouds. Proceedings of the 2016 IEEE 3rd World Forum on Internet of Things (WF-IoT).

[32] Pérez M.C., Gualda D., Vicente J., Villadangos J.M., Ureña J. Review of UAV positioning in indoor environments and new proposal based on US measurements. Proceedings of the 10th International Conference on Indoor Positioning and Indoor Navigation—Work-in-Progress (IPIN-WiP) Papers.

[33] De Croon G., De Wagter C. Challenges of Autonomous Flight in Indoor Environments. Proceedings of the 2018 IEEE/RSJ International Conference on Intelligent Robots and Systems (IROS).

[34] Bristeau P.-J., Callou F., Vissiere D., Petit N. (2011). The navigation and control technology inside the AR.Drone micro uav. IFAC Proc..

[35] Leutenegger S., Lynen S., Bosse M., Siegwart R., Furgale P. (2015). Keyframe-based visual–inertial odometry using nonlinear optimization. Int. J. Robot. Res..

[36] Tanskanen P., Naegeli T., Pollefeys M., Hilliges O. Semi-direct ekf-based monocular visual-inertial odometry. Proceedings of the 2015 IEEE/RSJ International Conference on Intelligent Robots and Systems (IROS).

[37] Balamurugan G., Valarmathi J., Naidu V.P.S. Survey on UAV Navigation in GPS Denied Environments. Proceedings of the 2016 International Conference on Signal Processing, Communication, Power and Embedded System.

[38] Shang C., Cheng L., Yu Q., Wang X., Peng R. Micro Aerial Vehicle Autonomous Flight Control in Tunnel Environment. Proceedings of the 9th International Conference on Modelling, Identification and Control (ICMIC).

[39] Paredes J.A., Álvarez F.J., Aguilera T., Villadangos J.M. (2018). 3D Indoor positioning of UAVs with spread spectrum ultrasound and Time-of-Flight Cameras. Sensors.

[40] Ashok A. Position: DroneVLC: Visible Light Communication for Aerial Vehicular Networking. Proceedings of the 4th ACM Workshop on Visible Light Communication Systems (VLCS).

[41] Opromolla R., Fasano G., Rufino G., Grassi M., Savvaris A. LIDAR-Inertial Integration for UAV Localization and Mapping in Complex Environments. Proceedings of the 2016 International Conference on Unmanned Aircraft Systems (ICUAS).

[42] Guerra E., Munguía R., Grau A. (2018). UAV Visual and Laser Sensor Fusion for Detection and Positioning in Industrial Applications. Sensors.

[43] Tiemann J., Wietfeld C. Scalable and Precise Multi-UAV Indoor Navigation using TDOA-based UWB Localization. Proceedings of the 2017 International Conference on Indoor Positioning and Indoor Navigation (IPIN).

[44] Perez-Grau F.J., Caballero F., Merino L., Viguria A. Multi-modal Mapping and Localization of Unmanned Aerial Robots based on Ultra-Wideband and RGB-D sensing. Proceedings of the 2017 IEEE/RSJ International Conference on Intelligent Robots and Systems (IROS).

[45] Li J., Bi Y., Li K., Wang K., Lin F., Chen B.M. Accurate 3D Localization for MAV Swarms by UWB and IMU Fusion. Proceedings of the 2018 IEEE 14th International Conference on Control and Automation (ICCA).

[46] Yan J., Yang X., Ye H., Wang Z. Integrated Positioning and Obstacle Avoidance for Multi-Quadrotors in an Indoor Office Environment. Proceedings of the 38th Chinese Control Conference.

[47] Macoir N., Bauwens J., Jooris B., Van Herbruggen B., Rossey J., Hoebeke J., De Poorter E. (2019). UWB Localization with Battery-Powered Wireless Backbone for Drone-Based Inventory Management. Sensors.

[48] Kwon W., Park J.H., Lee M., Her J., Kim S.-H., Seo J.-W. (2020). Robust Autonomous Navigation of Unmanned Aerial Vehicles (UAVs) for Warehouses’ Inventory Application. IEEE Robot. Autom. Lett..

[49] Mashood A., Dirir A., Hussein M., Noura H., Awwad F. Quadrotor object tracking using real-time motion sensing. Proceedings of the 5th International Conference on Electronic Devices, Systems and Applications (ICEDSA).

[50] Beul M., Droeschel D., Nieuwenhuisen M., Quenzel J., Houben S., Behnke S. (2018). Fast autonomous flight in warehouses for inventory applications. IEEE Robot. Autom. Lett..

[51] Campos-Macias L., Aldana-López R., de la Guardia R., Parra-Vilchis J.I., Gómez-Gutiérrez D. Autonomous Navigation of MAVs in Unknown Cluttered Environments. https://arxiv.org/abs/1906.08839.

[52] Welburn E., Hakim Khalili H., Gupta A., Watson S., Carrasco J. A navigational system for quadcopter remote inspection of offshore substations. Proceedings of the 15th International Conference on Autonomic and Autonomous Systems (ICAS).

[53] Kalinov I., Safronov E., Agishev R., Kurenkov M., Tsetserukou D. High-precision UAV localization system for landing on a mobile collaborative robot based on an IR marker pattern recognition. Proceedings of the 2019 IEEE 89th Vehicular Technology Conference (VTC2019-Spring).

[54] Särkkä S. (2013). Bayesian Filtering and Smoothing.

[55] Jazwinski A.H. (1970). Stochastic Processes and Filtering Theory.

[56] Gordon N.J., Salmond D.J., Smith A.F.M. (1993). Novel approach to nonlinear/non-Gaussian Bayesian state estimation. IEE Proc. F.

[57] Arulampalam S., Maskell S., Gordon N., Clapp T. (2002). A tutorial on particle filters for on-line non-linear/non-Gaussian Bayesian tracking. IEEE Trans. Signal Process..

[58] Gustafsson F., Gunnarsson F., Bergman N., Forssell U., Jansson J., Karlsson R., Nordlund P.J. (2002). Particle filters for positioning, navigation, and tracking. IEEE Trans. Signal Process..

[59] Simon D. (2006). Optimal State Estimation.

[60] Gustafsson F. (2010). Particle filter theory and practice with positioning applications. IEEE Aerosp. Electron. Syst. Mag..

[61] Del Moral P. (1996). Nonlinear filtering using random particles. Theory Probab. Appl..

[62] Hu X.L., Schon T.B., Ljung L. (2008). A basic convergence result for particle filtering. IEEE Trans Signal Process..

[63] Nordlund P.J., Gunnarsson F., Gustafsson F. Particle filters for positioning in wireless networks. Proceedings of the 11th European Signal Processing Conference (EUSIPCO).

[64] Li H., Wang J., Liu Y. Passive coherent radar tracking algorithm based on particle filter and multiple TDOA measurements. Proceedings of the 2nd International Congress on Image and Signal Processing (CISP).

[65] Boccadoro M., De Angelis G., Valigi P. (2012). TDOA positioning in NLOS scenarios by particle filtering. Wirel. Netw..

[66] Hol J.D., Schon T.B., Gustafsson F. On resampling algorithms for particle filters. Proceedings of the 2006 IEEE Nonlinear Statistical Signal Processing Workshop.

[67] Li T., Bolic M., Djuric P.M. (2015). Resampling methods for particle filtering: Classification, implementation, and strategies. IEEE Signal Process. Mag..

[68] Turgut B., Martin R.P. Restarting particle filters: An approach to improve the performance of dynamic indoor localization. Proceedings of the 2009 IEEE Global Telecommunications Conference (GLOBECOM).

[69] Guo K., Qiu Z., Miao C., Zaini A.H., Chen C.L., Meng W., Xie L. (2016). Ultra-Wideband-Based Localization for Quadcopter Navigation. Unmanned Syst..

[70] Tzoreff E., Weiss A.J. (2017). Expectation-maximization algorithm for direct position determination. Signal Process..

[71] Ma F., Yang L., Zhang M., Guo F.-C. (2019). TDOA source positioning in the presence of outliers. IET Signal Process..

